# 2,2′-Dihydroxybiphenyl-3,3′-di­carb­aldehyde dioxime

**DOI:** 10.1107/S1600536809029298

**Published:** 2009-07-29

**Authors:** Ekaterina Golovnia, Elena V. Prisyazhnaya, Turganbay S. Iskenderov, Matti Haukka, Igor O. Fritsky

**Affiliations:** a Kiev National Taras Shevchenko University, Department of Chemistry, Volodymyrska str. 64, 01601 Kiev, Ukraine; b Kyiv National University of Construction and Architecture, Department of Chemistry, Povitroflotsky Ave., 31, 03680 Kiev, Ukraine; c Karakalpakian University, Department of Chemistry, Universitet Keshesi 1, 742012 Nukus, Uzbekistan; d Department of Chemistry, University of Joensuu, PO Box 111, 80101 Joensuu, Finland

## Abstract

The mol­ecule of the title compound, C_14_H_12_N_2_O_4_, lies across a crystallographic inversion centre situated at the mid-point of the C—C intra-annular bond. The mol­ecule is not planar, the dihedral angle between the aromatic rings being 50.1 (1)°. The oxime group is in an *E* position with respect to the –OH group and forms an intra­molecular O—H⋯N hydrogen bond. In the crystal structure, inter­molecular O—H⋯O hydrogen bonds link mol­ecules into chains propagating along [001]. The crystal structure is further stabilized by inter­molecular stacking inter­actions between the rings [centroid-to-centroid distance = 3.93 (1) Å], resulting in layers parallel to the *bc* plane.

## Related literature

For the use of oximes as chelating ligands in coordination and analytical chemistry and extraction metallurgy, see: Kukushkin *et al.* (1996 ▶); Chaudhuri (2003 ▶). For the use of oxime ligands to obtain polynuclear compounds in the fields of mol­ecular magnetism and supra­molecular chemistry, see: Cervera *et al.* (1997 ▶); Costes *et al.* (1998 ▶). Oxime-containing ligands have been found to efficiently stabilize high oxidation states of metal ions such as Cu(III) and Ni(III), see: Fritsky *et al.* (2006 ▶); Kanderal *et al.* (2005 ▶). For C=N and N—O bond lengths in oximes, see: Mokhir *et al.* (2002 ▶); Onindo *et al.* (1995 ▶); Sliva *et al.* (1997 ▶). For the synthesis of 2,2′-dihydroxy­biphenyl-3,3′-dicarbaldehyde, see: Wünnemann *et al.* (2008 ▶).
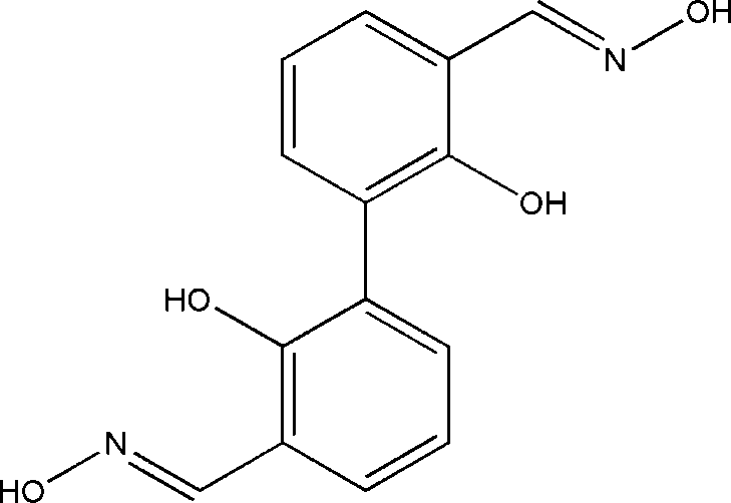


## Experimental

### 

#### Crystal data

C_14_H_12_N_2_O_4_
*M* *_r_* = 272.26
Monoclinic, 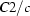

*a* = 24.2780 (14) Å
*b* = 3.9279 (4) Å
*c* = 16.6466 (12) Å
β = 129.652 (6)°
*V* = 1222.2 (2) Å^3^
*Z* = 4
Mo *K*α radiation
μ = 0.11 mm^−1^
*T* = 120 K
0.19 × 0.09 × 0.06 mm

#### Data collection

Nonius KappaCCD diffractometer
Absorption correction: multi-scan (*SADABS*; Sheldrick, 2001 ▶) *T*_min_ = 0.976, *T*_max_ = 0.993
4331 measured reflections
1388 independent reflections
812 reflections with *I* > 2σ(*I*)
*R*_int_ = 0.073

#### Refinement

*R*[*F* ^2^ > 2σ(*F* ^2^)] = 0.056
*wR*(*F* ^2^) = 0.146
*S* = 1.02
1388 reflections
99 parameters
H atoms treated by a mixture of independent and constrained refinement
Δρ_max_ = 0.27 e Å^−3^
Δρ_min_ = −0.29 e Å^−3^

### 

Data collection: *COLLECT* (Bruker–Nonius, 2004 ▶); cell refinement: *DENZO*/*SCALEPACK* (Otwinowski & Minor, 1997 ▶); data reduction: *DENZO*/*SCALEPACK*; program(s) used to solve structure: *SHELXS97* (Sheldrick, 2008 ▶); program(s) used to refine structure: *SHELXL97* (Sheldrick, 2008 ▶); molecular graphics: *DIAMOND* (Brandenburg, 2006 ▶); software used to prepare material for publication: *SHELXL97*.

## Supplementary Material

Crystal structure: contains datablocks global, I. DOI: 10.1107/S1600536809029298/jh2095sup1.cif

Structure factors: contains datablocks I. DOI: 10.1107/S1600536809029298/jh2095Isup2.hkl

Additional supplementary materials:  crystallographic information; 3D view; checkCIF report

## Figures and Tables

**Table 1:** Hydrogen-bond geometry (Å, °)

*D*—H⋯*A*	*D*—H	H⋯*A*	*D*⋯*A*	*D*—H⋯*A*
O1—H1⋯N1	0.91 (3)	1.79 (3)	2.609 (2)	148 (2)
O2—H2⋯O1^i^	1.00 (3)	1.96 (3)	2.871 (2)	151 (3)

Symmetry code: (i) 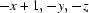.

**Fig. 1.**
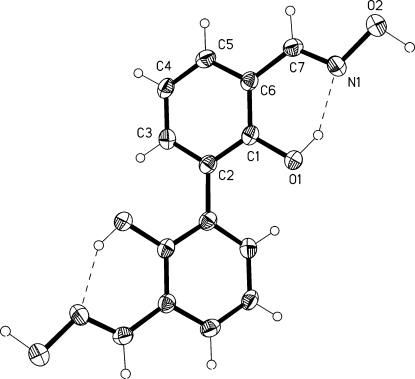
A view of compound (I), with displacement ellipsoids shown at the 50% probability level. H atoms are drawn as spheres of an arbitrary radius.

**Fig. 2.**
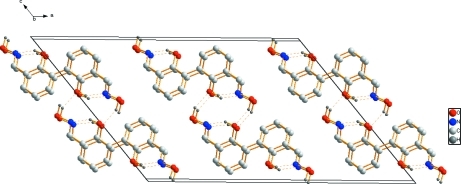
A packing diagram of the title compound. Hydrogen bonds are indicated by dashed lines. H atoms not involved in hydrogen bonding have been omitted for clarity.

## supplementary crystallographic information

### Comment

Oximes are a traditional class of chelating ligands widely used in coordination and analytical chemistry and extraction metallurgy (Kukushkin *et al.*, 1996; Chaudhuri, 2003). Due to marked ability to from bridges between metal ions oxime ligands may be used for obtaining polynuclear compounds in the field of molecular magnetism and supramolecular chemistry (Cervera *et al.*, 1997; Costes *et al.*, 1998). Also, the oxime ligands are strong donors and therefore the oxime-containing ligands were found to efficiently stabilize high oxidation states of metal ions like Cu(III) and Ni(III) (Kanderal *et al.*, 2005; Fritsky *et al.*, 2006). The presence of additional donor groups together with the oxime group in the ligand molecule may result in significant increase of chelating efficiency and ability to form polynuclear complexes. The present investigation is dedicated to the study of the molecular structure of the title compound (I) which is a new polynuclear ligand containing both oxime and phenolic functions.

Molecules of I lie across a crystallographic inversion centre situated in the midpoint of the C—C intra-annular bond (Fig. 1). The molecule is not planar, the dihedral angle between the phenyl rings is 50.1 (1)°. The oxime group is in the *E*-position with respect to the OH group and forms an intramolecular O—H···N hydrogen bond. The C=N and N—O bond lengths are normal for oximes (Onindo *et al.*, 1995; Sliva *et al.*, 1997; Mokhir *et al.*, 2002).

In the crystal structure, intermolecular O—H···O hydrogen bonds between the phenolic groups of the translational molecules link the molecules into chains propagating along [001]. The crystal structure is further stabilized by the intermolecular stacking interactions between the phenyl rings with centroid-to-centroid distances equal to 3.93 Å resulting in layers parallel to the *yz* plane (Fig. 2).

### Experimental

2,2'-Dihydroxybiphenyl-3,3'-dicarbaldehyde (2.57 g, 10 mmol) dissolved in 20 ml of methanol was added to a solution obtained by dissolving sodium (0.51 g, 22 mmol) in 10 ml of methanol with addition of hydroxylamine hydrochloride (1.52 g, 22 mmol). The mixture was stirred for 30 min and filtered. In 2–3 h the filtrate produced white crystalline precipitate which was filtered off and dried. Yield 85%. Single crystals suitable for X-ray analysis were obtained as a result of recrystallization from aqueous (40%) ethanol. 2,2'-Dihydroxybiphenyl-3,3'-dicarbaldehyde was synthesized according to the reported method (Wünnemann *et al.*, 2008).

### Refinement

The O—H hydrogen atoms were located from the difference Fourier map and refined isotropically. The C—H hydrogen atoms of the phenyl rings were positioned geometrically and were constrained to ride on their parent atoms, with C—H = 0.95 Å, and *U*_iso_ = 1.2 *U*_eq_(parent atom).

### Crystal data

**Table:**

C_14_H_12_N_2_O_4_	*F*(000) = 568
*M**_r_* = 272.26	*D*_x_ = 1.480 Mg m^−^^3^
Monoclinic, *C*2/*c*	Mo *K*α radiation, λ = 0.71073 Å
Hall symbol: -C 2yc	Cell parameters from 516 reflections
*a* = 24.2780 (14) Å	θ = 4.5–27.0°
*b* = 3.9279 (4) Å	µ = 0.11 mm^−^^1^
*c* = 16.6466 (12) Å	*T* = 120 K
β = 129.652 (6)°	Block, pale-yellow
*V* = 1222.2 (2) Å^3^	0.19 × 0.09 × 0.06 mm
*Z* = 4	

### Data collection

**Table:**

Nonius KappaCCD diffractometer	1388 independent reflections
Radiation source: fine-focus sealed tube	812 reflections with *I* > 2σ(*I*)
horizontally mounted graphite crystal	*R*_int_ = 0.073
Detector resolution: 9 pixels mm^-1^	θ_max_ = 27.5°, θ_min_ = 4.4°
φ scans and ω scans with κ offset	*h* = −30→30
Absorption correction: multi-scan (*SADABS*; Sheldrick, 2001)	*k* = −5→4
*T*_min_ = 0.976, *T*_max_ = 0.993	*l* = −18→21
4331 measured reflections	

### Refinement

**Table:**

Refinement on *F*^2^	Primary atom site location: structure-invariant direct methods
Least-squares matrix: full	Secondary atom site location: difference Fourier map
*R*[*F*^2^ > 2σ(*F*^2^)] = 0.056	Hydrogen site location: inferred from neighbouring sites
*wR*(*F*^2^) = 0.146	H atoms treated by a mixture of independent and constrained refinement
*S* = 1.02	*w* = 1/[σ^2^(*F*_o_^2^) + (0.0673*P*)^2^] where *P* = (*F*_o_^2^ + 2*F*_c_^2^)/3
1388 reflections	(Δ/σ)_max_ < 0.001
99 parameters	Δρ_max_ = 0.27 e Å^−^^3^
0 restraints	Δρ_min_ = −0.29 e Å^−^^3^

### Special details

**Table:**

Geometry. All e.s.d.'s (except the e.s.d. in the dihedral angle between two l.s. planes) are estimated using the full covariance matrix. The cell e.s.d.'s are taken into account individually in the estimation of e.s.d.'s in distances, angles and torsion angles; correlations between e.s.d.'s in cell parameters are only used when they are defined by crystal symmetry. An approximate (isotropic) treatment of cell e.s.d.'s is used for estimating e.s.d.'s involving l.s. planes.
Refinement. Refinement of *F*^2^ against ALL reflections. The weighted *R*-factor *wR* and goodness of fit *S* are based on *F*^2^, conventional *R*-factors *R* are based on *F*, with *F* set to zero for negative *F*^2^. The threshold expression of *F*^2^ > σ(*F*^2^) is used only for calculating *R*-factors(gt) *etc*. and is not relevant to the choice of reflections for refinement. *R*-factors based on *F*^2^ are statistically about twice as large as those based on *F*, and *R*- factors based on ALL data will be even larger.

### Fractional atomic coordinates and isotropic or equivalent isotropic displacement parameters (Å^2^)

**Table:**

	*x*	*y*	*z*	*U*_iso_*/*U*_eq_	
O1	0.50535 (8)	0.1656 (4)	0.11701 (11)	0.0286 (5)	
O2	0.64023 (9)	−0.1055 (4)	0.07166 (13)	0.0350 (5)	
N1	0.60748 (10)	0.0232 (5)	0.11062 (14)	0.0279 (5)	
C1	0.55751 (12)	0.2918 (5)	0.21487 (16)	0.0236 (6)	
C2	0.53803 (11)	0.4208 (6)	0.27199 (16)	0.0235 (6)	
C3	0.59205 (12)	0.5499 (6)	0.37151 (16)	0.0265 (6)	
H3	0.5795	0.6439	0.4105	0.032*	
C4	0.66275 (12)	0.5455 (6)	0.41490 (17)	0.0269 (6)	
H4	0.6983	0.6329	0.4832	0.032*	
C5	0.68185 (12)	0.4140 (6)	0.35911 (16)	0.0272 (6)	
H5	0.7308	0.4102	0.3893	0.033*	
C6	0.62978 (11)	0.2855 (6)	0.25813 (16)	0.0237 (6)	
C7	0.65242 (12)	0.1435 (6)	0.20269 (17)	0.0265 (6)	
H7	0.7019	0.1402	0.2358	0.032*	
H1	0.5270 (14)	0.081 (7)	0.0923 (19)	0.042 (8)*	
H2	0.5979 (18)	−0.165 (8)	−0.002 (3)	0.067 (9)*	

### Atomic displacement parameters (Å^2^)

**Table:**

	*U*^11^	*U*^22^	*U*^33^	*U*^12^	*U*^13^	*U*^23^
O1	0.0229 (9)	0.0387 (10)	0.0217 (9)	−0.0034 (7)	0.0132 (8)	−0.0059 (7)
O2	0.0324 (10)	0.0468 (11)	0.0296 (10)	0.0008 (8)	0.0217 (9)	−0.0042 (8)
N1	0.0299 (11)	0.0321 (11)	0.0277 (11)	0.0015 (9)	0.0212 (10)	−0.0001 (8)
C1	0.0244 (13)	0.0233 (12)	0.0192 (12)	−0.0006 (9)	0.0121 (11)	0.0013 (9)
C2	0.0235 (12)	0.0218 (12)	0.0213 (11)	−0.0002 (9)	0.0124 (11)	0.0017 (9)
C3	0.0306 (14)	0.0266 (13)	0.0231 (12)	−0.0015 (10)	0.0176 (11)	0.0000 (10)
C4	0.0253 (13)	0.0301 (13)	0.0178 (11)	−0.0044 (10)	0.0103 (10)	−0.0024 (9)
C5	0.0211 (12)	0.0290 (14)	0.0257 (12)	−0.0018 (10)	0.0123 (11)	0.0011 (10)
C6	0.0237 (13)	0.0246 (12)	0.0204 (12)	−0.0012 (9)	0.0130 (11)	0.0020 (9)
C7	0.0207 (12)	0.0311 (13)	0.0252 (12)	−0.0008 (10)	0.0136 (11)	0.0008 (10)

### Geometric parameters (Å, °)

**Table:**

O1—C1	1.368 (3)	C3—C4	1.373 (3)
O1—H1	0.91 (3)	C3—H3	0.9500
O2—N1	1.402 (2)	C4—C5	1.376 (3)
O2—H2	1.00 (3)	C4—H4	0.9500
N1—C7	1.276 (3)	C5—C6	1.402 (3)
C1—C2	1.399 (3)	C5—H5	0.9500
C1—C6	1.409 (3)	C6—C7	1.453 (3)
C2—C3	1.396 (3)	C7—H7	0.9500
C2—C2^i^	1.490 (4)		
			
C1—O1—H1	107.9 (16)	C3—C4—C5	119.7 (2)
N1—O2—H2	101.8 (18)	C3—C4—H4	120.1
C7—N1—O2	112.73 (17)	C5—C4—H4	120.1
O1—C1—C2	118.89 (19)	C4—C5—C6	120.7 (2)
O1—C1—C6	120.46 (19)	C4—C5—H5	119.7
C2—C1—C6	120.6 (2)	C6—C5—H5	119.7
C3—C2—C1	118.0 (2)	C5—C6—C1	118.83 (19)
C3—C2—C2^i^	120.9 (2)	C5—C6—C7	118.8 (2)
C1—C2—C2^i^	121.1 (2)	C1—C6—C7	122.31 (19)
C4—C3—C2	122.1 (2)	N1—C7—C6	121.6 (2)
C4—C3—H3	118.9	N1—C7—H7	119.2
C2—C3—H3	118.9	C6—C7—H7	119.2
			
O1—C1—C2—C3	−179.69 (18)	C4—C5—C6—C7	−178.9 (2)
C6—C1—C2—C3	1.6 (3)	O1—C1—C6—C5	−179.3 (2)
O1—C1—C2—C2^i^	0.3 (3)	C2—C1—C6—C5	−0.6 (3)
C6—C1—C2—C2^i^	−178.47 (16)	O1—C1—C6—C7	−0.8 (3)
C1—C2—C3—C4	−1.7 (3)	C2—C1—C6—C7	177.9 (2)
C2^i^—C2—C3—C4	178.39 (17)	O2—N1—C7—C6	−179.16 (18)
C2—C3—C4—C5	0.8 (3)	C5—C6—C7—N1	−179.9 (2)
C3—C4—C5—C6	0.3 (3)	C1—C6—C7—N1	1.5 (3)
C4—C5—C6—C1	−0.3 (3)		

Symmetry codes: (i) −*x*+1, *y*, −*z*+1/2.

### Hydrogen-bond geometry (Å, °)

**Table:**

*D*—H···*A*	*D*—H	H···*A*	*D*···*A*	*D*—H···*A*
O1—H1···N1	0.91 (3)	1.79 (3)	2.609 (2)	148 (2)
O2—H2···O1^ii^	1.00 (3)	1.96 (3)	2.871 (2)	151 (3)

Symmetry codes: (ii) −*x*+1, −*y*, −*z*.
